# The positive impact of a care–physical activity initiative for people with a low socioeconomic status on health, quality of life and societal participation: a mixed-methods study

**DOI:** 10.1186/s12889-022-13936-w

**Published:** 2022-08-10

**Authors:** Lisanne Sofie Mulderij, Kirsten T. Verkooijen, Stef Groenewoud, Maria A. Koelen, Annemarie Wagemakers

**Affiliations:** 1 Health and Society Group, Wageningen University & Research, P.O. Box 8130, 6700 EW Wageningen, the Netherlands; 2 Radboud University Medical Centre, Radboud Institute for Health Sciences, Scientific Centre for Quality of Healthcare, IQ healthcare, P.O. Box 9101, 6500 HB Nijmegen, the Netherlands

**Keywords:** Lifestyle intervention, Physical activity, Low socioeconomic status, Health promotion, Overweight, Obesity

## Abstract

**Background:**

Overweight and obesity rates are increasing worldwide, particularly among people with a low socioeconomic status (SES). Care–physical activity (care–PA) initiatives may improve participants’ lifestyles and thereby lower overweight and obesity rates. A two-year care–PA initiative specifically developed for citizens with a low SES, X-Fittt 2.0, was offered free of charge to participants, and included 12 weeks of intensive guidance and sports sessions, and 21 months of aftercare. Here, we study the impact of X-Fittt 2.0 on health, quality of life (QoL) and societal participation using a mixed-methods design.

**Methods:**

Questionnaires and body measurements were taken from 208 participants at the start of X-Fittt 2.0 (t_0_) and after 12 weeks (t_1_), one year (t_2_) and two to three years (t_3_). We also held 17 group discussions (t_1_, *n* = 71) and 68 semi-structured interviews (t_2_ and t_3_). Continuous variables were analysed using a linear mixed-model analysis (corrected for gender, age at t_0_, height, education level and employment status at the different time points), while we used descriptive statistics for the categorical variables. Qualitative data were analysed using a thematic analysis.

**Results:**

Body weight was significantly lower at all three post-initiative time points compared with the baseline, with a maximum of 3.8 kg difference at t_2_. Body Mass Index, waist circumference, blood pressure and self-perceived health only significantly improved during the first 12 weeks. A positive trend regarding paid work was observed, while social visits decreased. The latter might be explained by the COVID-19 pandemic, as lockdowns limited social life. Furthermore, participants reported increased PA (including sports) and a few stopped smoking or drinking alcohol. Participants mentioned feeling healthier, fitter and more energetic. Additionally, participants’ self-esteem and stress levels improved, stimulating them to become more socially active. However, the participants also mentioned barriers to being physically active, such as a lack of money or time, or physical or mental health problems.

**Conclusions:**

X-Fittt 2.0 improved the health, QoL and societal participation of the participants. Future initiatives should take into account the aforementioned barriers, and consider a longer intervention period for more sustainable results. More complete data are needed to confirm the findings.

**Supplementary Information:**

The online version contains supplementary material available at 10.1186/s12889-022-13936-w.

## Background

Overweight and obesity rates are increasing worldwide [1]. In the Netherlands, the percentage of adults who were overweight increased from 32% in 1990 to 49% in 2015, and these rates are expected to rise in the coming decades [2]. Being overweight or obese is also more prevalent among people with a low socioeconomic status (SES) than for people with a higher SES [3]; for instance, 19.8% of the low-educated Dutch adults were obese in 2015, compared with ‘only’ 8.4% of the high educated Dutch population [2]. This higher prevalence of obesity shows that there are health inequalities between people with a low SES and people with a higher SES in the Netherlands, which is also prevalent worldwide [2–4]. In addition to the difference in overweight and obesity rates, the difference in life expectancy and years in good perceived health is indicative of these health inequalities in the Netherlands; for example, people with a low education level are expected to live 7 years less than people with a high education level, and with 18 fewer years in good perceived health [2]. Furthermore, people with a low SES generally score worse for self-perceived health and quality of life (QoL) than people with a higher SES [5–7]. Lastly, the higher prevalence of chronic diseases (e.g., obesity and diabetes) among people with a low SES might also lead to lower societal participation among this group [8]. To tackle this overweight and obesity problem and to reduce health inequalities, care–physical activity (care–PA) initiatives have been developed. The impact of such initiatives on people with a low SES has, however, not yet been studied.

In care–PA initiatives, professionals from the healthcare sector (e.g., lifestyle coach, physiotherapist, and dietitian) and the PA sector (i.e., sports coaches) work together to improve the health and lifestyle of citizens and to reduce the risk of developing chronic diseases by increasing daily PA and improving dietary behaviours [9, 10]. PA is known to be able to increase fitness, QoL, self-esteem and stress levels; to reduce the symptoms of depression or anxiety and the risk of developing chronic diseases; and to improve social skills, societal participation and employment status [11–14]. However, because people with a low SES experience specific problems, such as stress due to poverty, debts or unemployment, they are often hard to reach for participation in (lifestyle) interventions, either by the health care sector or the sports sector [15]. It is therefore crucial to tailor care–PA initiatives specifically to people with a low SES by minimising the barriers to participate in these initiatives, such as language and literacy barriers and financial barriers, and by providing the intervention close to the homes of the participants [16–18]. Furthermore, coaches should use a more personal and intensive (e.g., longer) approach, be committed to the target population and use behaviour change techniques such as goal setting [16–19].

This study evaluates the impact of a Dutch care–PA initiative developed specifically for people with a low SES: X-Fittt 2.0. For this initiative, a low SES was determined as an income at or below the minimum wage level and receiving municipal benefits [20]. Our research question was: what are the short- and long-term outcomes of participation in X-Fittt 2.0 in terms of health, QoL, and societal participation? We expected that X-Fittt 2.0 is capable of positively influencing participants’ health and QoL by improving their lifestyle. Furthermore, we expected that, because of the positive influences on health and QoL, participants will participate more in society (e.g., work, voluntary work or other social activities).

### X-Fittt 2.0

X-Fittt 2.0 has been specifically developed for people with a low SES by a sports centre, a Dutch municipality and a healthcare insurance company [21]. Participation in the initiative was free of charge and comprised a 12-week intensive programme at a local sports centre, involving three parts: weekly sports sessions (two in a group with a sports coach, one individually) consisting of a combination of strength exercises and fitness training; dietary advice and monitoring by a dietician; and 4 h of coaching by a qualified and higher educated (Bachelor level) lifestyle coach who supported the participants to work on their personal goals. After these 12 weeks, the participants received aftercare over a period of approximately 21 months, comprising a total of 6 h of lifestyle coaching to encourage behavioural maintenance. X-Fittt 2.0 was conducted at different sport centres in two municipalities in the Netherlands under different names, but for clarity we will refer to it as X-Fittt 2.0 in the present paper. Both sport centres collaborated with the researchers to provide the quantitative data and to recruit participants for group discussions and individual interviews. Although no formal fidelity assessment was conducted, the interviews with participants and informal chats with program deliverers (lifestyle coaches, sport coaches) suggested that the quality of program delivery was largely as described in the X-Fittt 2.0 protocol. The experiences of X-Fittt 2.0 participants with the program are described elsewhere [22].

## Methods

### Data collection

**Fig. 1:**
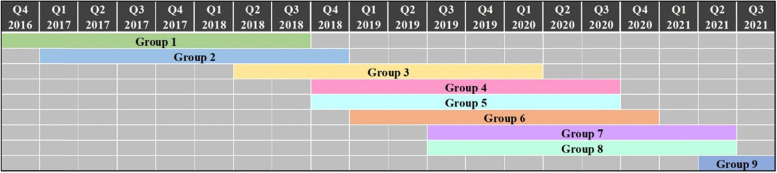
Timeline of groups that started X-Fittt 2.0

This mixed-methods study evaluated an existing care–PA initiative in which the quantitative and qualitative data collection ran parallel and integration took place at the interpretation and reporting level [23, 24]. The justification for the chosen outcome variables (Table 1), which have also been used in other studies evaluating care–PA initiatives [27–30], can be found in our study protocol [9]. We studied nine groups participating in X-Fittt 2.0 (*n* = 208) over a period of 5 years, from 2016 to 2021 (Fig. 1). Each group started with the 12-week intensive programme. Questionnaires and body measurements were taken at the start (t_0_), after 12 weeks (t_1_), after one year (t_2_) and after two to three years (t_3_) (Table 1). The questionnaires were distributed by the lifestyle coaches connected to X-Fittt 2.0, while the body measurements were performed by physiotherapists at the sports centres where X-Fittt 2.0 was conducted. After the first two groups that we studied, we adapted the questionnaires, because it appeared that participants did not understand some of the questions. The problematic sets of questions explored the confidence participants had in maintaining PA and participants’ motivation to be physically active but were too difficult for the participants to understand, and we could not rely on them being completed properly. We decided to remove them from the questionnaires and instead added questions concerning societal participation, because these were missing in the first version of the questionnaires. Hence, the data for the outcome variable societal participation were incomplete for the first two groups who underwent X-Fittt 2.0.

**Table 1:** Overview of the data collection methods, measurements and number of participants at each time point

Collection method	Measurements	***n***			
		t_**0**_	t_**1**_	t_**2**_	t_**3**_
Questionnaire	QoL score and self-perceived health (EQ-5D-3L and EQ-VAS [25]), illness and medicine use, visits to healthcare professionals, monitoring of PA, societal participation (USER-P [26]), smoking and alcohol behaviour, demographic information	144	101	79	42
Body measurements	Height, body weight, body mass index (BMI), fat percentage, waist circumference, blood pressure	169	117	58	38
Group discussion	Health, daily life, societal participation, group experience and group dynamics, motivation, continuation of sports	–	71	–	–
Individual interview	Health, daily life, societal participation, sports participation and PA, nutritional behaviour	–	–	31	37

X-Fittt 2.0 comprised a two-year programme, but because of the COVID-19 pandemic, measurements at t_3_ could not always be taken at the end of the programme. The measurements for group 1 and 2 were taken after 2 years, but the final measurements for the other groups were taken between two and 3 years after the start of X-Fittt 2.0. All participants were assigned a unique study ID that could only be traced back to the participant by the researchers.

At t_1_, group discussions were held with the participants. The topics of these group discussions were participants’ health, diet, daily life, motivation, experiences with the group, societal participation and whether the participants would continue to exercise after the first 12 weeks. After 16 group discussions, data saturation was reached and only one additional already planned discussion was conducted. At t_2_ and t_3_, we held semi-structured interviews, in which we asked participants about their health, diet, PA and societal participation. After about 34 interviews both at t_2_ and t_3_, data saturation was reached and only the interviews that were already planned were conducted.

All methods were carried out in accordance with relevant guidelines and regulations.

### Data analysis

#### Body measurements and questionnaires

**Fig. 2:**
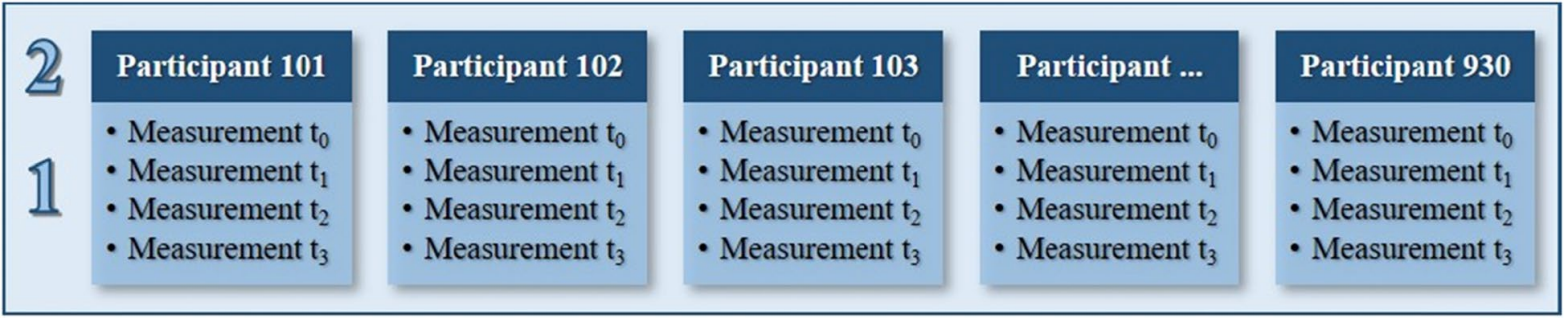
The two levels of the multilevel model used in this study

We used a linear mixed-model analysis with a maximum likelihood estimation to assess the changes over time (repeated measurements) using a first-order autoregressive covariance structure with heterogenous variances. The primary outcomes included body weight, Body Mass Index (BMI), waist circumference, blood pressure, QoL and self-perceived health. The basic model consisted of an outcome measure, time as repeated measures (first level) and the participants’ study IDs (second level) (Fig. 2). We then step by step included gender, age at t_0_, height, education level and employment status at t_0_ as second-level fixed factors, and employment status at different time points as a first-level fixed factor. Subsequent models were compared in terms of the likelihood ratio test and Schwarz’s Bayesian information criterion (BIC) (Additional file 1). Data were included for all participants who finished at least the first 12 weeks of X-Fittt 2.0. We compared all estimated marginal means with the baseline measurements (t_0_) and the significance was assessed at *a* = .05. For categorical variables, we used descriptive statistics, meaning that we calculated frequencies for all time points separately. No statistical tests were performed. All analyses of the quantitative data were performed using IBM SPSS Statistics, version 25.0.

#### Group discussions and individual interviews

We conducted a thematic analysis, which consisted of the six steps described by Braun & Clarke (2006) [31]. All steps were conducted separately for the group discussions at t_1_ and the individual interviews at t_2_ and t_3_. After familiarisation with the data (step 1), we inductively generated initial codes by reading the data (step 2). About 50% of the data were coded by two (interviews at t_2_ and t_3_) or three (group discussions at t_1_) researchers, after which we discussed the encodings of the different researchers. Because the researchers had coded the data quite similarly, only one researcher coded the rest of the data. Data saturation was reached after coding approximately 75% of the group discussions and individual interviews, as no new codes were applied. After all data were coded, we sorted the codes into potential themes (step 3). We then reviewed the themes by reading the data extracts within each theme (step 4). During this step, some initial themes were merged, and some codes or data extracts were moved to another theme (Additional file 2). Also, relevant data extracts that were missed during the initial coding were coded. After that, we defined and further refined the themes and analysed their content by identifying the essence of each theme separately and of all themes together (step 5). This is also the step in which we named each of the three final themes that we developed for both the group discussions and the individual interviews: *‘lifestyle’*, *‘self-perceived health’* and *‘societal participation’*. Finally, we wrote down the results and selected accompanying data extracts to be presented in the paper (step 6). All analyses of the qualitative data were performed using ATLAS.ti, version 9.

## Results

### Body measurements and questionnaires

#### Participant characteristics

In total, 181 participants completed at least the first 12 weeks of X-Fittt 2.0, of whom three quarters were women and about 20 % were educated to a low level (Table 2). About one third of the group did not work (paid or voluntary). More than one quarter of the participants were overweight and almost two third of the participants were obese. Twenty-seven participants dropped out during the first 12 weeks or at an unspecified moment later on. The only observed difference between these groups was that, compared with the dropouts, the participant group was slightly more highly educated and more commonly participated in paid or voluntary work.

**Table 2:** Baseline measurements for participants and dropouts as measured at t_0_

	Participants *n = 181*			Dropouts *n = 27*		
	***n***	Mean (SD)	***n*** (%)	***n***	Mean (SD)	***n*** (%)
**Age (years)**	165	48.2 (11.9)		20	44.8 (10.7)	
**Gender**	179			25		
Female			128 (71.5)			18 (72.0)
Male			51 (28.5)			7 (28.0)
**Education level**^**a**^	138			16		
Low			28 (20.3)			2 (25.0)
Middle			88 (63.8)			11 (68.8)
High			22 (15.9)			1 (6.3)
**Work status**	144			18		
Paid/voluntary work			92 (63.9)			9 (56.3)
No paid/voluntary work			52 (36.1)			7 (43.8)
**Height (cm)**	167	168.0 (9.2)		21	169.1 (12.3)	
**Weight (kg)**	169	92.3 (19.6)		22	96.3 (18.2)	
**BMI (kg/m**^**2**^**)**	165	32.7 (5.7)		21	33.0 (3.7)	
Healthy weight			10 (6.1)			0 (0.0)
Overweight			49 (29.7)			4 (19.0)
Obese			106 (64.2)			17 (81.0)
**Waist circumference (cm)**	166	107.8 (15.5)		23	110.2 (12.8)	
**Blood pressure (mmHg)**	167			23		
Systolic		131.4 (18.9)			127.77 (15.5)	
Diastolic		83.5 (12.5)			80.1 (11.3)	
**Self-perceived health**^**b**^	144	6.2 (1.6)		16	6.1 (1.5)	

^a^ **Low education level:** leaving after primary school, preparatory secondary vocational education, senior secondary vocational education level one, or the first three years of senior general secondary education or pre–university education; **middle education level:** leaving after completing senior general secondary education or pre–university education, or senior secondary vocational education level two, three or four; **high education level:** completed higher professional education or university [32]

^b^ EQ–VAS: scale from 0 to 10 [25]

The main self-reported reasons for dropout were the development of injuries, physical or mental health problems, an intense life event or lack of time. Furthermore, some of the participants could no longer be reached by the lifestyle coaches and were therefore classified as dropout.

#### Physical health and QoL

Eventually, the final model was corrected for gender, age at t_0_, height, education level and employment status at different time points (Additional file 3). Compared with the start of X-Fittt 2.0, the participants lost an average of 2.6 kg of weight during the first intensive 12 weeks (Table 3). After 1 year, this average weight loss had grown to about 3.8 kg. Although the participants gained some weight during the second year of X-Fittt 2.0, they still maintained an average loss of 3.4 kg compared with the start of the initiative. BMI, waist circumference and blood pressure was only significantly decreased at t_1_ compared with the start. Self-perceived health increased significantly by about 0.6 (on a scale of 0 to 10) during the first 12 weeks. No significant changes were observed for the other variables or for the other time points.

**Table 3:** Estimated marginal means and average changes for the different time points for all outcome measures

	Participants *n = 181*								
	t_**0**_	t_**1**_	t_**2**_	t_**3**_	t_**1**_ – t_**0**_	t_**2**_ – t_**0**_	t_**2**_ – t_**1**_	t_**3**_ – t_**0**_	t_**3**_ – t_**2**_
**Weight**^**a**^	96.0	93.3	92.2	92.6	– 2.64*	– 3.84*	– 1.19	– 3.42*	– 0.42
**BMI**^**b**^	34.0	33.2	32.8	32.9	– 0.85*	– 1.24	– 0.39	– 1.08	+ 0.16
**Waist circumference**^**c**^	111.1	107.3	108.5	110.1	– 3.75*	– 2.57	+ 1.19	– 1.02	+ 1.54
**Systolic blood pressure**^**d**^	131.2	125.0	130.6	130.4	– 6.25*	– 0.57	+ 5.68	– 0.78	– 0.21
**Diastolic blood pressure**^**d**^	83.2	80.3	80.3	81.9	– 2.85*	– 2.84	+ 0.01	– 1.22	+ 1.62
**Quality of life**^**e**^	0.68	0.65	0.65	0.63	– 0.03	– 0.03	+ 0.00	– 0.04	– 0.02
**Self-perceived health**^**f**^	6.2	6.9	6.5	6.6	+ 0.65*	+ 0.27	– 0.38	+ 0.38	+ 0.11

^a^ kilograms; ^b^ kilograms/m^2^; ^c^ centimetres; ^d^ mmHg; ^e^ EQ–5D–3 L: scale from – 0.33 to 1.00 [25]; ^f^ EQ–VAS: scale from 0 to 10 [25]; * *p* < 0.05

#### Societal participation and lifestyle

Regarding societal participation, we saw a trend in the data for paid work (Additional file 4). At the start of X-Fittt 2.0, the vast majority of the participants did not have a paid job. At the end of X-Fittt 2.0 (t_3_), this was still the case, although a few participants had started paid work or worked more hours per week compared with the start of X-Fittt 2.0. On the other hand, participants reported visiting others or receiving visitors slightly less often than at the baseline. This could, however, be explained by the COVID-19 pandemic, during which lockdowns have limited social life in the Netherlands.

For 15 participants we had data on lifestyle both at t_0_ and at t_3_. These data show that the number of participants who did not exercise decreased (from 5 to 2) over the course of X-Fittt 2.0, while the number of people who exercised regularly increased compared with the start of X-Fittt 2.0 (from 10 to 13) (Additional file 4). For PA in a non–organised sports setting (walking, cycling, etc.), the number of active people had increased, especially at t_1_ and t_2,_ compared with t_0_. Furthermore, more people monitored their own PA behaviour (Additional file 5). No changes in the use of medicines were observed. Of the participants who had a measurement both at t_0_ and t_2_ (*n* = 39), two stopped smoking and seven stopped drinking alcohol over the course of X-Fittt 2.0.

### Group discussions (GD) and individual interviews (I)

#### Lifestyle

Participants indicated that they were more aware of healthy lifestyles and healthy nutrition. They watched their diet more and tried to snack less, and the smokers indicated smoking less. *“Yes, now instead of tobacco, I have an e-cigarette, but I am at such a level now that I have almost completely stopped. That is because I am working out here [at the sports centre of X-Fittt 2.0].”* [GD7 – t_1_] Furthermore, they had obtained new knowledge and their mindset had changed, which helped many of them to maintain the healthy lifestyle after the first 12 weeks of X-Fittt 2.0.

Some participants, though, found maintaining a healthy lifestyle difficult (mentioned in 9 interviews at t_2_ and in 17 interviews at t_3_). This was especially true for PA, for which they mentioned several reasons. First, a lack of money was seen as a major barrier to continue with sports after the first 12 weeks of X-Fittt 2.0. *“People who have enough money may not understand it. Like ‘if you want to work out, just go and work out’. But if you cannot afford it... I really need it [to work out].”* [GD9 – t_1_] *“I am paying off debt, so a gym subscription is out of the question.”* [I11 – t_3_] Second, having to do it on their own, a lack of discipline or having problems picking it up again was mentioned as barrier. *“They closed for a week because new equipment was installed and things changed. Then I could not work out for 14 days, it all went to pot, and at some point I just stopped going.”* [GD5 – t_1_] Third, a lack of time, for instance due to an increase in (voluntary) work activities, hampered being physically active. *“I really do feel like ‘I really want this [to exercise]’, but I have a lot of work assignments at the moment and it sort of slips by.”* [I10 – t_3_] Fourth, the physical and mental health of the participants was perceived as a barrier. *“And my [physical] health is actually deteriorating more and more. More and more complaints, which I didn’t have then. So that’s why I have done less and less, actually.”* [I14 – t_3_] *“Psychiatric problems reared their heads and as a result I could not keep that commitment [of exercising]”* [I21 – t_3_] Fifth, feeling tired or already having exercised resulted in less PA among participants. *“I used to go walking at the weekend, but now I often think ‘I’ve already done my workout on Wednesday and Thursday’, so I’m not going for a walk at the weekend, even though I really enjoy it.”* [I9 – t_2_] Finally, the COVID-19 pandemic, during which sports centres were closed for a few months, made it difficult for participants to exercise. *“I can’t exercise now. Or rather, I don’t exercise. I can, but I don’t exercise because I’m a bit scared of catching the virus.”* [I43 – t_3_].

Although some participants had trouble maintaining a healthy lifestyle, a myriad of the participants reported an increase in PA since participation in X-Fittt 2.0 (mentioned in 5 group discussions at t_1_, 17 interviews at t_2_ and 18 interviews at t_3_). Many of them also mentioned that they did not exercise at all before the start of X-Fittt 2.0. Although about half of them continued to exercise at a sports centre or sports club after the first intensive 12 weeks, others stopped doing this. Of this last group, most participants indicated spending their leisure time on more active pursuits. *“I have a dog. Before I started I used to do small laps and now I just walk and then I think ‘I can do another lap’. My dog totally loves it. It doesn’t take any energy to walk, whereas before it did.”* [I7 – t_2_] Two participants at t_2_ and 3 participants at t_3_ mentioned that they had relapsed into less physically active behaviours during the 2 years of X-Fittt 2.0, but that they were able to resume their healthier lifestyle and to be physically active again.

#### Self-perceived health

Some participants experienced negative effects of participating in X-Fittt 2.0, especially after the first 12 weeks, such as tiredness and injuries caused by sports (mentioned in 6 group discussions). At t_2_ (*n* = 1) and t_3_ (*n* = 5), a few participants reported having more physical problems than before the start of X-Fittt 2.0, which they related to exercising. *“But that is the other side of the story. After the fifth group meeting I injured my back. Since then, I have not participated in any groups at [the sports centre]. I went through a long process of physiotherapy. [I thought to myself,] ‘If only I had not started doing sports, if only I had not joined [X-Fittt 2.0]’.”* [I30 – t_2_] Participants who were less physically active after the intensive first 12 weeks indicated that, after t_1_, their stamina decreased and that they had more psychological problems, were more tired, and had gained weight (discussed in 7 interviews at t_2_ and t_3_). Two participants mentioned that the COVID-19 pandemic had also played a part in this.

However, the majority of the participants indicated experiencing an improved health status at t_1_ (mentioned in 13 group discussions), but also one to 3 years after the start of X-Fittt 2.0 (*n* = 26 at t_2_ and *n* = 33 at t_3_). For instance, they felt fitter, have more energy and have an improved stamina. *“Yes, I have a 5-year-old daughter. Before I started this, I was exhausted after an hour with her, and now I can spend a whole afternoon with her.”* [GD1 – t_1_] It was also mentioned that their body measurements had improved (e.g., decreased body weight, fat percentage, abdominal circumference and blood pressure), and that they became stronger and more *in shape*. Furthermore, participants mentioned reductions in the amount of pain and physical problems they experienced, and in their use of medication (mentioned in 2 group discussions at t_1_, 5 interviews at t_2_ and 6 interviews at t_3_). *“But when you see that I can now walk 15 kilometres... Last week I went to [my vascular surgeon] for my annual check-up. ‘I never thought you would ever manage that with your leg’, he said.”* [I48 – t_3_] Some participants indicated improvements in their mental health, such as experiencing less stress or feeling mentally stronger (mentioned in 7 group discussions at t_1_, 12 interviews at t_2_ and 8 interviews at t_3_). *“But also in terms of resilience, I feel strengthened.”* [GD7 – t_1_] Additionally, they also mentioned improvements in self-esteem. *“And seeing that you lose weight every week, that you keep to the right eating schedule and so on, that has given me a lot of self-confidence in daily life.”* [GD5 – t_1_] *“It’s more of a general feeling of being more comfortable in my own skin. I did feel really uncomfortable and unattractive ... So that shame is actually gone, and that makes a difference.”* [I7 – t_2_].

#### Societal participation

The majority of the participants indicated a positive impact of X-Fittt 2.0 on their daily activities, because they feel fitter and have more energy (mentioned in 6 group discussions at t_1_, 8 interviews at t_2_ and 9 interviews at t_3_). For instance, participating in X-Fittt 2.0 gave participants structure in their daily life. Two participants explained at t_3_ that before participating in X-Fittt 2.0, they used to lie on the sofa and they felt socially isolated, but that they are now fitter and therefore more assertive in undertaking activities. *“Wanting more, being able to do more. Before, you had to pull me out of the house, so to speak. Now I am getting out of the house myself, and I come up with things and initiatives.”* [GD7 – t_1_]. Furthermore, a few participants mentioned that they work more or started working again after a period of not working (both for paid work and voluntary work) (mentioned in 1 group discussion at t_1_, 2 interviews at t_2_ and 4 interviews at t_3_). An increase in self-esteem and energy levels had contributed to this, according to the participants. On the other hand, some participants mentioned that they undertook fewer activities due to tiredness or muscular soreness caused by sports (mentioned in 1 group discussion at t_1_, 3 interviews at t_2_ and 2 interviews at t_3_). Another reason mentioned was that it was difficult to combine daily activities with the sports sessions, especially during the first 12 weeks of X-Fittt 2.0 when they attended two sports sessions a week.

The sports sessions in the first 12 weeks were seen as a social activity, during which the participants could socialise with others. *“I was active before that too, but still, at a certain point I missed those social things. Because you sit at home. Being among people for a while did some good.”* [I5 – t_2_] Others indicated feeling less lonely 1 year after the start of X-Fittt 2.0 and participating in more social activities (*n* = 12 at t_2_). *“I am more likely to say yes if someone says ‘come with me to Ikea’ or something like that. Before, I would have said no, but now I say yes... Because you are fitter, you can do it. And you are not constantly thinking about how embarrassing it is that you are so fat.”* [I7 – t_2_].

A few participants had hoped that participation in X-Fittt 2.0 would boost their social life, allowing them to make new friends with the other group members, but for them that did not happen (mentioned in 3 interviews at t2 and 1 interview at t3). *“I still see two of them occasionally–we do the same group lesson–but otherwise not. I had a goal to get to know more people, but that hasn’t happened yet. In the future, I might get to know more people outside the gym, but not yet.”* [I9 – t_2_].

At t_3_, few participants mentioned aspects regarding their social life. Two participants felt the need for a romantic relationship, while a three others indicated that they are satisfied with their social life, even though some of them would have liked to have more social contacts. Three others mentioned that their number of social contacts had improved over the few past years. *“I came through a very difficult phase during which I locked myself up very much, because I had to get better first. Then I got [X-Fittt 2.0] and I felt very good. And then I dared to go out on the street more often. I go out to dinner with friends, we have card nights, we go out. And yes, I really started living a different life.”* [I20 – t_3_].

## Discussion

We studied a care–PA initiative that was specifically developed for people with a low SES, investigating its impact on the participants’ health, QoL and societal participation. We followed the participants for a period of two to 3 years, which enabled us to study the impact of such an initiative on the short term as well as the long term. This study shows that X-Fittt 2.0 was able to significantly reduce participants’ body weight at all time points, and that waist circumference, blood pressure and self-perceived health decreased significantly during the first 12 weeks of the programme. Weight loss was highest 1 year after the start of X-Fittt 2.0. Participants also mentioned that they felt healthier, less stressed and more confident after participating in X-Fittt 2.0. Furthermore, positive trends were observed during the 2 years of X-Fittt 2.0 regarding paid work, sports and PA, and smoking and alcohol behaviour. Several participants also indicated that they have increased their daily activities, for instance because they feel more confident and have more energy. These results are in line with previous studies that show that PA is associated with an improved physical and mental health status, higher QoL levels, higher self-esteem and lower stress levels [11–14].

To obtain a complete picture of the impact of care–PA initiatives, a narrative integration of quantitative and qualitative data is valuable [23]. The qualitative results of this study support, and to a lesser degree expand, the quantitative results that we observed. For instance, complementary to the observed health gains in the body measurements and questionnaires, during the group discussions and interviews many participants highlighted the positive impact of X-Fittt 2.0 on their physical health, such as weight loss, having more energy and having fewer physical complaints. Furthermore, increases in self-perceived health during the first 12 weeks, as measured with the questionnaires, were supported by changes in self-esteem and stress levels that many participants mentioned not only during the group discussions, but also in the interviews at t_2_ and t_3_. In addition, the data showed positive trends regarding paid work, which was supported by a few participants who indicated during the group discussions and interviews that they had worked more or started a paid job during the 2 years of X-Fittt 2.0. The quantitative results also showed that more people started sports or increased their PA in another way, which was supported by a myriad of the participants mentioning that they started exercising during X-Fittt 2.0, despite not being physically active at all before. Although some participants stopped sports after t_1_, they indicated during the interviews that they had increased their PA outside the sports setting. Nevertheless, some participants who stopped being physically active indicated that they felt less healthy in the period after the first 12 weeks, which might explain the absence of statistically significant changes in self-perceived health after t_1_. In this way, the qualitative results helped to gain a better understanding of the contextual factors that may have influenced the quantitative results. Furthermore, although we did not observe a quantitative difference in the use of medication, a few participants indicated during the group discussions and interviews that they had been able to reduce the amount of medication they took, for instance for diabetes or pain relief. Lastly, the positive trends in smoking and alcohol behaviour in the quantitative data were supported by the qualitative data. Thus, although statistical significance was missing in the quantitative data for most outcomes, the qualitative results supported the clinical relevance of X-Fittt 2.0.

QoL, as measured by the EQ-5D-3L, was rather stable throughout the study, whereas self-perceived health, measured with the EQ-VAS, increased significantly during the first 12 weeks. During 6 group discussions and 6 interviews, participants indicated that they experienced more physical problems and pain (due to injuries and muscle soreness), but that they still rated their health as higher overall because of improvements in other aspects, such as improved mental health. This could explain the difference we found in QoL and self-perceived health as measured using the questionnaires; however, these are speculations and more research is needed to support this.

Although a comparison with other care–PA initiatives is difficult because not all care–PA initiatives consist of the same programme components and involve different study populations, X-Fittt 2.0 seems to be equally successful, or even more successful, when compared with other care–PA initiatives, both for the general population and for people with a low SES. For instance, our results with regards to weight loss are better than previously studied care–PA initiatives, and although participants gained a bit of weight back after 1 year, the weight loss we observed at t_2_ and t_3_ was still larger than in the other studies [27–30]. Furthermore, the improvements in waist circumference and self-perceived health during the first 12 weeks are comparable with these other care–PA initiatives, as are improvements in sports and PA levels [27–30]. Despite these positive results, the absence of sustained positive results in our study could be caused by the decrease in the programme intensity after the first 12 weeks. The outcomes might have been more positive in the long term if the initial period of intensive guidance and sports activities had lasted longer than 12 weeks.

### Strengths and limitations

An important strength of our study is the use of a mixed-methods design. Mixed-methods studies generally produce a more complete picture of a certain topic to inform theory and practice, and can be used to improve the generalisability of the results [33]. In our study, the use of mixed methods resulted in triangulation, with body measurements, self-reported measurements and qualitative data from the group discussions and interviews being used to obtain a complete and credible overview of health outcomes [23, 24, 34]. In addition, we reached data saturation with the group discussions (*n* = 71) and 68 individual interviews, ensuring that our qualitative data was reliable and adds to the body of knowledge on the impact of care–PA initiatives.

Another strength is the longitudinal character of our study, which helped to elucidate the impact of the initiative on the participants not only in the short term, but also over a longer period. This is important, as it highlights whether the results of X-Fittt 2.0 are maintained in the years after the intensive 12-week programme. We were able to collect and analyse data at two and 3 years after the start of X-Fittt 2.0 (t_3_), collected via questionnaires (*n* = 42) and body measurements (*n* = 38) for the quantitative data and via interviews (*n* = 37) for the qualitative data, which gave us insight into the long-term effects. In addition, the real-life setting of our study may have increased its external validity compared with a controlled design. Furthermore, people with a low SES can be hard to reach with research projects, but our collaboration with an existing care–PA initiative positively influenced the number of participants that we could include in our study. Due to this collaboration, the results obtained during the study period were immediately valuable to practice. Lastly, although we experienced some missing data, the type of data analysis that we used handles this issue relatively well [35]. With the use of this analysis method, we were able to include the data of all participants, instead of only the data from those participants who completed measurements at least two of the time points.

Some limitations should be mentioned as well. Although the chosen statistical analysis handles missing data quite well [35], the high amount of missing data in our study (55%) is still a major limitation. There are several explanations for this large amount of missing data. First, most of the data were not collected by the researchers themselves, but by lifestyle coaches and physiotherapists connected to X-Fittt 2.0. We believe that the distribution of the questionnaires by the lifestyle coaches had a positive impact on our response rate, because all participants had regular meetings with the lifestyle coaches; however, for the body measurements, a separate appointment with a physiotherapist was made, often at a later date, and not all participants attended. We think that this explains the lower number of completed body measurements at t_2_ compared with the number of completed questionnaires at that time. We recommend that future researchers ensure that questionnaires and body measurements are taken at the same time. Second, X-Fittt 2.0 lasted for 2 years, but was not equally intensive throughout those 2 years. During the first 12 weeks, the participants had meetings with a lifestyle coach every one or 2 weeks. After those 12 weeks, this decreased to every few months, which made it harder to stay in touch with the participants, and hampered the collection of follow-up data for many of them. Third, not everyone in the group of participants spoke Dutch equally well, which made it hard or even impossible for some participants to fill out the questionnaires. The lifestyle coaches also indicated that the meetings they had with those participants were rather ‘*useless*’, because they did not have the feeling that their messages regarding a healthy lifestyle were understood. Finally, the COVID-19 pandemic has influenced data collection because not all participants were willing to visit the sports centres for their follow-up measurements at t_3_ during the pandemic. Furthermore, the pandemic might have influenced participants’ health behaviours and responses to our questionnaires and interviews.

Another limitation of our study is that the body measurements were not performed by the same person nor at the same location at each time point. This means that the equipment that was used differed among the locations; for instance, at some locations, fat percentage was measured using skinfold thickness, while at other locations a weighing scale with built-in fat meter was used. Furthermore, skinfold thickness as measurement of fat percentage is prone to intra-observer error [36]. Thus, the different measuring persons or measurement locations made it difficult to compare the results, and we decided to exclude fat percentage from the outcome measures. Nevertheless, bias could still be present for the other body measurements. Future research should aim to minimise bias by creating a comprehensive research protocol and by using the same person and equipment for all measurements.

## Conclusions

We aimed to study care–PA initiatives developed specifically for citizens with low SES, investigating their impact on the health, QoL, and societal participation of the participants. Although some participants felt less healthy after participating in X-Fittt 2.0 due to injuries or feeling tired, the majority of the participants experienced an improved health status. Participants lost weight, had more stamina, felt fitter and less stressed, reported a higher self-esteem and rated their own health more highly, especially immediately after the intensive 12-week period of the programme. Furthermore, participation in work increased for a few participants, and some mentioned undertaking more social and day-to-day activities. They also exercised more and generally had higher PA levels outside the sports setting. Overall, their awareness of a healthy lifestyle had increased, which improved their lifestyle. The participants mentioned several barriers to being physically active however, such as a lack of money or time, physical or mental health problems, and the COVID-19 pandemic. Thus, the intensive guidance at the start of the programme stimulated the participants to live more healthily and improved their health in the short term, but their independent continuation of the healthy lifestyle appeared more difficult for participants with a lack of resources. Future initiatives should aim to find ways to minimise the mentioned barriers and consider a longer intervention period. Furthermore, future research should aim for a more complete data collection to confirm the results reported here.

## Supplementary Information

**Additional file 1 **Schwarz’s Bayesian information criterion, chi-square values and *p*-values for the basic model, intermediate models and final model.

**Additional file 2.** Codes and themes identified throughout the steps of the thematic analysis.

**Additional file 3.** Estimates of the intercept and fixed effects of the final models.

**Additional file 4.** Quantitative data for societal participation.

**Additional file 5.** Quantitative data for lifestyle behaviour.

## Data Availability

The dataset generated and analysed during the current study is available in the DANS-EASY repository: 10.17026/dans-24c-q6rh.

## Abbreviations

BMI: Body mass index
Care–PA initiative: Care–physical activity initiative
GD: Group discussions
I: Individual interviews
SES: Socioeconomic status
QoL: Quality of life
