# Supplementary material for: The positive impact of a care–physical activity initiative for people with a low socioeconomic status on health, quality of life and societal participation: a mixed-methods study
Source: BMC Public Health. 2022 Aug 10;22:1522. doi: 10.1186/s12889-022-13936-w (PMC9363851; doi:10.1186/s12889-022-13936-w)
Supplement: Supplementary file 1 — Additional file 1 Schwarz’s Bayesian information criterion, chi-square values and p-values for the basic model, intermediate models and final model. [file 12889_2022_13936_MOESM1_ESM.pdf]

Additional file 1: Schwarz’s Bayesian information criterion, chi-square values and p-values for the basic model, intermediate models and final model

Table 1.1 Basic model, intermediate models and final model for body weight

Model	Schwarz’s Bayesian Criterion (BIC)	df	Chi-square	p-value
Gender, Time, Age, Height	2751.6	12	–	–
Gender, Time, Age, Height, Education level	2352.6	17	–399.0	<.00001
Gender, Time, Age, Height, Education level, Employment status (8 categories)	1925.6	24	–427.0	<.00001
Gender, Time, Age, Height, Education level, Employment status (2 categories) (final model)	1894.8	18	–30.8	<.00001

Table 1.2 Basic model, intermediate models and final model for BMI

Model	Schwarz’s Bayesian Criterion (BIC)	df	Chi-square	p-value
Gender, Time, Age, Height	2006.4	12	–	–
Gender, Time, Age, Height, Education level	1726.5	17	–279.9	<.00001
Gender, Time, Age, Height, Education level, Employment status (8 categories)	1463.0	24	–263.5	<.00001
Gender, Time, Age, Height, Education level, Employment status (2 categories) (final model)	1432.1	18	–30.9	<.00001

Table 1.1 Basic model, intermediate models and final model for waist circumference

Model	Schwarz’s Bayesian Criterion (BIC)	df	Chi-square	p-value
Gender, Time, Age, Height	2620.4	12	–	–
Gender, Time, Age, Height, Education level	2233.6	17	–386.7	<.00001
Gender, Time, Age, Height, Education level, Employment status (8 categories)	1911.2	24	–322.4	<.00001
Gender, Time, Age, Height, Education level, Employment status (2 categories) (final model)	1880.0	18	–31.3	<.00001

Table 1.2 Basic model, intermediate models and final model for systolic blood pressure

Model	Schwarz’s Bayesian Criterion (BIC)	df	Chi-square	p-value
Gender, Time, Age, Height	3004.9	12	–	–
Gender, Time, Age, Height, Education level	2578.7	17	–426.2	<.00001
Gender, Time, Age, Height, Education level, Employment status (8 categories)	2076.6	24	–502.1	<.00001
Gender, Time, Age, Height, Education level, Employment status (2 categories) (final model)	2050.0	18	–26.6	<.00001

Table 1.3 Basic model, intermediate models and final model for diastolic blood pressure

Model	Schwarz's Bayesian Criterion (BIC)	df	Chi-square	p-value
Gender, Time, Age, Height	2717.3	12	–	–
Gender, Time, Age, Height, Education level	2321.4	17	–395.9	<.00001
Gender, Time, Age, Height, Education level, Employment status (8 categories)	1884.1	24	–437.3	<.00001
Gender, Time, Age, Height, Education level, Employment status (2 categories) (final model)	1853.3	18	–30.8	<.00001

Table 1.4 Basic model, intermediate models and final model for QoL

Model	Schwarz's Bayesian Criterion (BIC)	df	Chi-square	p-value
Gender, Time, Age, Height	91.4	12	–	–
Gender, Time, Age, Height, Education level	112.4	17	21.1	<.001
Gender, Time, Age, Height, Education level, Employment status (8 categories)	150.8	24	38.3	<.00001
Gender, Time, Age, Height, Education level, Employment status (2 categories) (final model)	124.1	18	–26.7	<.00001

Table 1.5 Basic model, intermediate models and final model for self–rated health

Model	Schwarz's Bayesian Criterion (BIC)	df	Chi-square	p-value
Gender, Time, Age, Height	1236.9	12	–	–
Gender, Time, Age, Height, Education level	1170.2	17	–66.7	<.00001
Gender, Time, Age, Height, Education level, Employment status (8 categories)	1070.6	24	–99.6	<.00001
Gender, Time, Age, Height, Education level, Employment status (2 categories) (final model)	1042.8	18	–27.8	<.00001
