# Supplementary material for: The positive impact of a care–physical activity initiative for people with a low socioeconomic status on health, quality of life and societal participation: a mixed-methods study
Source: BMC Public Health. 2022 Aug 10;22:1522. doi: 10.1186/s12889-022-13936-w (PMC9363851; doi:10.1186/s12889-022-13936-w)
Supplement: Supplementary file 2 — Additional file 2. Codes and themes identified throughout the steps of the thematic analysis. [file 12889_2022_13936_MOESM2_ESM.pdf]

Additional file 2: codes and themes identified throughout the steps of the thematic analysis

Table 2.1 Codes and themes for group discussions identified through the steps of the thematic analysis.

Codes step 2	Themes step 3	Themes step 4	Themes step 5
Awareness	Behaviour	Behaviour/Lifestyle	Lifestyle and behaviour
Behaviour change – positive	Behaviour	Behaviour/Lifestyle	Lifestyle and behaviour
Daily activities – negative	Daily life	Daily life	Daily life
Daily activities – neutral	Daily life	Daily life	Daily life
Daily activities – positive	Daily life	Daily life	Daily life
Employment	Daily life	Daily life	Daily life
Effect – negative	Health	Health	Health
Effect – neutral	Health	Health	Health
Effect – positive	Health	Health	Health
Lifestyle	Lifestyle	Behaviour/Lifestyle	Lifestyle and behaviour
Motivation – positive	Behaviour	Behaviour/Lifestyle	Lifestyle and behaviour
Physical activity	Lifestyle	Behaviour/Lifestyle	Lifestyle and behaviour
Sports	Lifestyle	Behaviour/Lifestyle	Lifestyle and behaviour
Structure	Daily life	Deleted, data extracts not relevant	Deleted, data extracts not relevant

Table 2.2 Codes and themes for individual interviews identified through the steps of the thematic analysis.

Codes step 2	Themes step 3	Themes step 4	Themes step 5
Awareness	Behaviour	Behaviour/Lifestyle	Lifestyle and behaviour
Behaviour change	Behaviour	Behaviour/Lifestyle	Lifestyle and behaviour
Daily activities	Daily life	Daily life	Daily life
Daily activities – negative	Daily life	Daily life	Daily life
Daily activities – neutral	Daily life	Daily life	Daily life
Daily activities – positive	Daily life	Daily life	Daily life
Effect – negative	Health	Health	Health
Effect – neutral	Health	Health	Health
Effect – positive	Health	Health	Health
Health – mental	Health	Health	Health
Health – physical	Health	Health	Health
Lifestyle	Lifestyle	Behaviour/Lifestyle	Lifestyle and behaviour
Motivation	Behaviour	Behaviour/Lifestyle	Lifestyle and behaviour
Nutrition	Lifestyle	Behaviour/Lifestyle	Lifestyle and behaviour
Physical activity	Lifestyle	Behaviour/Lifestyle	Lifestyle and behaviour
Smoking behaviour	Lifestyle	Behaviour/Lifestyle	Lifestyle and behaviour
Social life – score	Social life	Social life	Social life
Social life – negative	Social life	Social life	Social life
Social life – neutral	Social life	Social life	Social life
Social life – positive	Social life	Social life	Social life
Sports	Lifestyle	Behaviour/Lifestyle	Lifestyle and behaviour
Support	Social life	Social life	Social life
