# Supplementary material for: The positive impact of a care–physical activity initiative for people with a low socioeconomic status on health, quality of life and societal participation: a mixed-methods study
Source: BMC Public Health. 2022 Aug 10;22:1522. doi: 10.1186/s12889-022-13936-w (PMC9363851; doi:10.1186/s12889-022-13936-w)
Supplement: Supplementary file 3 — Additional file 3. Estimates of the intercept and fixed effects of the final models. [file 12889_2022_13936_MOESM3_ESM.pdf]

Additional file 3: estimates of the intercept and fixed effects of the final models

Table 3.1 Estimates of the intercept and fixed effects for body weight

Final model	Estimate	Std. Error	p-value
Intercept	-56.010	35.159	0.114
Fixed part			
Gender (male)	-0.057	4.000	0.989
Gender (female)	reference		
Education level 0	-8.158	9.310	0.382
Education level 1	-2.552	9.519	0.789
Education level 2	-8.745	8.421	0.301
Education level 3	-5.412	9.118	0.554
Education level 4	-6.337	10.874	0.561
Education level 5	reference		
Employment status 0	2.065	1.046	0.051
Employment status 1	reference		
Time 1	3.420	1.582	0.035
Time 2	0.778	1.572	0.623
Time 3	-0.415	1.645	0.802
Time 4	reference		
Age at start	-0.402	0.121	0.001
Height	1.022	0.202	0.000

Table 3.2 Estimates of the intercept and fixed effects for BMI

Final model	Estimate	Std. Error	p-value
Intercept	50.924	11.573	0.000
Fixed part			
Gender (male)	0.525	1.301	0.687
Gender (female)	reference		
Education level 0	-3.207	3.113	0.305
Education level 1	-0.439	3.174	0.890
Education level 2	-3.548	2.813	0.209
Education level 3	-1.220	3.028	0.688
Education level 4	-2.265	3.649	0.536
Education level 5	reference		
Employment status 0	0.877	0.453	0.055
Employment status 1	reference		
Time 1	1.083	0.646	0.100
Time 2	0.231	0.651	0.724
Time 3	-0.159	0.867	0.855
Time 4	reference		
Age	-0.147	0.040	0.000
Height	-0.058	0.067	0.386

Table 3.3 Estimates of the intercept and fixed effects for waist circumference

Final model	Estimate	Std. Error	p-value
Intercept	48.374	28.175	0.089
Fixed part			
Gender (male)	-1.623	3.248	0.618
Gender (female)	reference		
Education level 0	-7.556	7.465	0.313
Education level 1	-2.532	7.645	0.741
Education level 2	-7.459	6.722	0.269
Education level 3	-8.094	7.279	0.268
Education level 4	-2.417	8.795	0.784
Education level 5	reference		
Employment status 0	2.437	1.336	0.070
Employment status 1	reference		
Time 1	1.024	1.798	0.571
Time 2	-2.727	1.849	0.145
Time 3	-1.542	1.973	0.437
Time 4	reference		
Age	-0.131	0.098	0.185
Height	0.429	0.163	0.010

Table 3.4 Estimates of the intercept and fixed effects for systolic blood pressure

Final model	Estimate	Std. Error	p-value
Intercept	127.506	30.323	0.000
Fixed part			
Gender (male)	9.837	3.441	0.005
Gender (female)	reference		
Education level 0	2.033	8.154	0.804
Education level 1	3.670	8.304	0.660
Education level 2	7.994	7.209	0.270
Education level 3	7.339	7.842	0.352
Education level 4	-5.744	9.643	0.553
Education level 5	reference		
Employment status 0	-0.244	2.229	0.913
Employment status 1	reference		
Time 1	0.779	2.988	0.795
Time 2	-5.475	3.172	0.089
Time 3	0.206	3.242	0.950
Time 4	reference		
Age	0.263	0.106	0.015
Height	-0.102	0.175	0.563

Table 3.5 Estimates of the intercept and fixed effects for diastolic blood pressure

Final model	Estimate	Std. Error	p-value
Intercept	55.944	20.941	0.009
Fixed part			
Gender (male)	1.358	2.372	0.568
Gender (female)	reference		
Education level 0	6.786	5.721	0.238
Education level 1	8.796	5.751	0.130
Education level 2	10.358	5.011	0.042
Education level 3	12.055	5.450	0.029
Education level 4	10.921	6.611	0.101
Education level 5	reference		
Employment status 0	-0.111	1.460	0.940
Employment status 1	reference		
Time 1	1.221	2.101	0.564
Time 2	-1.632	2.124	0.446
Time 3	-1.616	2.190	0.464
Time 4	reference		
Age	-0.052	0.073	0.478
Height	0.117	0.121	0.336

Table 3.6 Estimates of the intercept and fixed effects for QoL

Final model	Estimate	Std. Error	p-value
Intercept	1.353	0.500	0.008
Fixed part			
Gender (male)	0.132	0.056	0.020
Gender (female)	reference		
Education level 0	-0.058	0.135	0.668
Education level 1	-0.092	0.137	0.504
Education level 2	0.043	0.121	0.721
Education level 3	-0.033	0.130	0.800
Education level 4	0.039	0.157	0.802
Education level 5	reference		
Employment status 0	-0.083	0.033	0.011
Employment status 1	reference		
Time 1	0.042	0.043	0.334
Time 2	0.015	0.048	0.755
Time 3	0.017	0.045	0.711
Time 4	reference		
Age	0.000	0.002	0.962
Height	-0.004	0.003	0.134

Table 3.7 Estimates of the intercept and fixed effects for self-rated health

Final model	Estimate	Std. Error	p-value
Intercept	4.284	2.653	0.109
Fixed part			
Gender (male)	0.008	0.298	0.979
Gender (female)	reference		
Education level 0	-0.612	0.729	0.403
Education level 1	-0.694	0.740	0.350
Education level 2	-0.247	0.651	0.704
Education level 3	-0.414	0.699	0.554
Education level 4	-0.061	0.827	0.941
Education level 5	reference		
Employment status 0	-0.268	0.170	0.117
Employment status 1	reference		
Time 1	-0.381	0.239	0.117
Time 2	0.265	0.244	0.283
Time 3	-0.113	0.254	0.656
Time 4	reference		
Age	0.029	0.009	0.003
Height	0.008	0.015	0.589
