# Supplementary material for: The positive impact of a care–physical activity initiative for people with a low socioeconomic status on health, quality of life and societal participation: a mixed-methods study
Source: BMC Public Health. 2022 Aug 10;22:1522. doi: 10.1186/s12889-022-13936-w (PMC9363851; doi:10.1186/s12889-022-13936-w)
Supplement: Supplementary file 4 — Additional file 4. Quantitative data for societal participation. [file 12889_2022_13936_MOESM4_ESM.pdf]

Additional file 4: societal participation

Table 4.1 Frequencies for participants who had a measurement both at t_0 and t_1 (n = 57).

	t_0: n (%)	t_1: n (%)
Paid work		
Not	53 (93.0)	50 (87.7)
< 17 hours/week	0 (0.0)	1 (1.8)
≥ 17 hours/week	4 (7.0)	6 (10.5)
Voluntary work		
Not	29 (50.9)	27 (47.4)
< 17 hours/week	26 (45.6)	27 (47.4)
≥ 17 hours/week	2 (3.5)	3 (5.3)
Education		
Not	47 (82.5)	51 (89.5)
< 17 hours/week	9 (15.8)	6 (10.5)
≥ 17 hours/week	1 (1.8)	0 (0.0)
Household chores		
Not	2 (3.5)	1 (1.8)
< 17 hours/week	37 (64.9)	39 (68.4)
≥ 17 hours/week	18 (31.6)	17 (29.8)
Sports		
Not	20 (35.1)	3 (5.3)
1x or less per week	7 (12.3)	7 (12.3)
Few times a week (2–4)	25 (43.9)	37 (64.9)
5 times a week or more	5 (8.8)	10 (17.5)
PA		
Not	6 (10.5)	5 (8.8)
1x or less per week	8 (14.0)	5 (8.8)
Few times a week (2–4)	25 (43.9)	21 (36.8)
5 times a week or more	18 (31.6)	26 (45.6)
Receive visitors		
Not	16 (28.1)	18 (31.6)
1x or less per week	24 (42.1)	31 (54.4)
Few times a week (2–4)	16 (28.1)	8 (14.0)
5 times a week or more	1 (1.8)	0 (0.0)
Visit others		
Not	9 (15.8)	9 (15.8)
1x or less per week	24 (42.1)	29 (50.9)
Few times a week (2–4)	22 (38.6)	17 (29.8)
5 times a week or more	2 (3.5)	2 (3.5)

Table 4.2 Frequencies for participants who had a measurement both t_0 and t_2 (n = 39).

	t_0: n (%)	t_2: n (%)
Paid work		
Not	34 (87.2)	29 (74.4)
< 17 hours/week	2 (5.1)	2 (5.1)
≥ 17 hours/week	3 (7.7)	8 (20.5)
Voluntary work		
Not	23 (59.0)	27 (69.2)
< 17 hours/week	16 (41.0)	12 (30.8)
≥ 17 hours/week	0 (0.0)	0 (0.0)
Education		
Not	33 (84.6)	31 (79.5)
< 17 hours/week	5 (12.8)	7 (17.9)
≥ 17 hours/week	1 (2.6)	1 (2.6)
Household chores		
Not	1 (2.6)	0 (0.0)
< 17 hours/week	28 (71.8)	30 (76.9)
≥ 17 hours/week	10 (25.6)	9 (23.1)
Sports		
Not	16 (41.0)	8 (20.5)
1x or less per week	3 (7.7)	11 (28.2)
Few times a week (2–4)	17 (43.6)	13 (33.3)
5 times a week or more	3 (7.7)	7 (17.9)
PA		
Not	2 (5.1)	4 (10.3)
1x or less per week	5 (12.8)	1 (2.6)
Few times a week (2–4)	17 (43.6)	9 (23.1)
5 times a week or more	15 (38.5)	25 (64.1)
Receive visitors		
Not	10 (25.6)	9 (23.1)
1x or less per week	19 (48.7)	25 (64.1)
Few times a week (2–4)	10 (25.6)	5 (12.8)
5 times a week or more	0 (0.0)	0 (0.0)
Visit others		
Not	5 (12.8)	10 (25.6)
1x or less per week	20 (51.3)	19 (48.7)
Few times a week (2–4)	13 (33.3)	8 (20.5)
5 times a week or more	1 (2.6)	2 (5.1)

Table 4.3 Frequencies for participants who had a measurement both at t_0 and t_3 (n = 15).

	t_0: n (%)	t_3: n (%)
Paid work		
Not	14 (93.3)	10 (66.7)
< 17 hours/week	0 (0.0)	2 (13.3)
≥ 17 hours/week	1 (6.7)	3 (20.0)
Voluntary work		
Not	9 (60.0)	8 (53.3)
< 17 hours/week	5 (33.3)	5 (33.3)
≥ 17 hours/week	1 (6.7)	2 (13.3)
Education		
Not	12 (80.0)	13 (86.7)
< 17 hours/week	3 (20.0)	2 (13.3)
≥ 17 hours/week	0 (0.0)	0 (0.0)
Household chores		
Not	1 (6.7)	0 (0.0)
< 17 hours/week	10 (66.7)	10 (66.7)
≥ 17 hours/week	4 (26.7)	5 (33.3)
Sports		
Not	5 (33.3)	2 (13.3)
1x or less per week	2 (13.3)	1 (6.7)
Few times a week (2–4)	7 (46.7)	7 (46.7)
5 times a week or more	1 (6.7)	5 (33.3)
PA		
Not	0 (0.0)	1 (6.7)
1x or less per week	2 (13.3)	3 (20.0)
Few times a week (2–4)	8 (53.3)	5 (33.3)
5 times a week or more	5 (33.3)	6 (40.0)
Receive visitors		
Not	3 (20.0)	4 (26.7)
1x or less per week	8 (53.3)	8 (53.3)
Few times a week (2–4)	4 (26.7)	2 (13.3)
5 times a week or more	0 (0.0)	1 (6.7)
Visit others		
Not	1 (6.7)	2 (13.3)
1x or less per week	8 (53.3)	9 (60.0)
Few times a week (2–4)	5 (33.3)	2 (13.3)
5 times a week or more	1 (6.7)	2 (13.3)
