# Supplementary material for: The positive impact of a care–physical activity initiative for people with a low socioeconomic status on health, quality of life and societal participation: a mixed-methods study
Source: BMC Public Health. 2022 Aug 10;22:1522. doi: 10.1186/s12889-022-13936-w (PMC9363851; doi:10.1186/s12889-022-13936-w)
Supplement: Supplementary file 5 — Additional file 5. Quantitative data for lifestyle behaviour. [file 12889_2022_13936_MOESM5_ESM.pdf]

Additional file 5: lifestyle behaviour

Table 5.1 Frequencies for participants who had a measurement both at t_0 and t_1 (n = 55).

	t_0: n (%)	t_1: n (%)
Monitoring PA behaviour		
No	43 (78.2)	39 (70.9)
Yes	12 (21.8)	16 (29.1)
Use of medicines		
No	12 (21.8)	9 (16.4)
Yes	43 (78.2)	46 (83.6)
Smoking		
No	33 (60.0)	33 (60.0)
Yes	22 (40.0)	22 (40.0)
Drinking alcohol (regularly)		
No	28 (50.9)	33 (60.0)
Yes	27 (49.1)	22 (40.0)

Table 5.2 Frequencies for participants who had a measurement both t_0 and t_2 (n = 39).

	t_0: n (%)	t_2: n (%)
Monitoring PA behaviour		
No	34 (87.2)	24 (61.5)
Yes	5 (12.8)	15 (38.5)
Use of medicines		
No	8 (20.5)	8 (20.5)
Yes	31 (79.5)	31 (79.5)
Smoking		
No	26 (66.7)	28 (71.8)
Yes	13 (33.3)	11 (28.2)
Drinking alcohol (regularly)		
No	23 (59.0)	31 (79.5)
Yes	16 (41.0)	8 (20.5)

Table 5.3 Frequencies for participants who had a measurement both at t_0 and t_3 (n = 12).

	t_0: n (%)	t_3: n (%)
Monitoring PA behaviour		
No	10 (83.3)	3 (25.0)
Yes	2 (16.7)	9 (75.0)
Use of medicines		
No	2 (16.7)	2 (16.7)
Yes	10 (83.3)	10 (83.3)
Smoking		
No	6 (50.0)	7 (58.3)
Yes	6 (50.0)	5 (41.7)
Drinking alcohol (regularly)		
No	5 (41.7)	7 (58.3)
Yes	7 (58.3)	5 (41.7)
